# Fungal Diversity and Aflatoxins in Maize and Rice Grains and Cassava-Based Flour (Pupuru) from Ondo State, Nigeria

**DOI:** 10.3390/jof7080635

**Published:** 2021-08-04

**Authors:** Daniella O. Ekpakpale, Bart Kraak, Martin Meijer, Kolawole I. Ayeni, Jos Houbraken, Chibundu N. Ezekiel

**Affiliations:** 1 Department of Microbiology, Babcock University, Ilishan Remo 121103, Ogun State, Nigeria; daniellegemscott@gmail.com (D.O.E.); kolaay2@gmail.com (K.I.A.); 2 Westerdijk Fungal Biodiversity Institute, Uppsalalaan, 3584 Utrecht, The Netherlands; b.kraak@wi.knaw.nl (B.K.); m.meijer@wi.knaw.nl (M.M.); j.houbraken@wi.knaw.nl (J.H.)

**Keywords:** aflatoxin, food safety, maize, mycology, Nigeria, pupuru, rice

## Abstract

Grains and cassava-based foods serve as major dietary sources for many households in Nigeria. However, these foods are highly prone to contamination by moulds and aflatoxins owing to poor storage and vending practices. Therefore, we studied the fungal diversity in maize, cassava-based flour (pupuru), and rice vended in markets from Ondo state, Nigeria, and assessed their aflatoxin levels using an enzyme-linked immunosorbent assay. Molecular analysis of 65 representative fungal isolates recovered from the ground grains and pupuru samples revealed 26 species belonging to five genera: *Aspergillus* (80.9%), *Penicillium* (15.4%), and *Talaromyces* (1.9%) in the *Ascomycota*; *Syncephalastrum* (1.2%) and *Lichtheimia* (0.6%) in *Mucoromycota*. *Aspergillus flavus* was the predominant species in the ground grains and pupuru samples. Aflatoxins were found in 73.8% of the 42 representative food samples and 41.9% exceeded the 10 μg/kg threshold adopted in Nigeria for total aflatoxins.

## 1. Introduction

Filamentous fungi contaminate food crops worldwide, thereby contributing significantly to the problem of food safety and food insecurity [1]. In tropical countries, e.g., Nigeria, favourable warm-to-hot climatic conditions coupled with poor pre- and post-harvest agricultural practices encourage widespread filamentous fungal contamination of grains, such as maize and rice [2,3,4], and cassava products (e.g., pupuru) [5]. Unlike maize and rice that are consumed and available worldwide, pupuru is a cassava-based, traditionally processed staple commonly consumed in south-west Nigeria. Pupuru processing includes the steeping of peeled cassava tubers in water for 4–5 days for submerged fermentation. The wet mash is bagged and dewatered using a mechanical press. The resulting fibres are handpicked from the mash prior to heating in an open fire, dry-milled, and sieved into pupuru flour [5,6]. Maize, rice, and pupuru principally contain carbohydrates (74 g, 80 g, and 94.6 g) and less amounts of protein (9.4 g, 7.1 g, and 2.9 g) and fibre (7.3 g, 1.3 g, and 0.9 g), respectively [7]. Together, these foods contribute significantly to the calorie intake of several households in Nigeria, with the specificity of pupuru to the Ondo and other south-western Nigerian population.

Maize, rice, and pupuru are commonly contaminated by fungal spores during pre- and post-harvest agricultural processing stages [8,9]. In Nigeria, vending of maize, rice, and pupuru in open markets could further expose these foods to fungal contamination [9]. Specifically, maize and rice are vended in open basins and plastic bowls, whereas pupuru is vended in covered basins and plastic bowls. Only a few studies used sequence data for identification of the fungi present on stored grains in Nigeria. A study of the mycobiota of maize [3] applied a robust polyphasic approach, and an investigation of vended rice used sequences of the internal transcribed spacer (ITS) region [4]. However, ITS sequencing is less suitable for species-level identification of common food-related genera such as *Aspergillus*, *Penicillium,* and *Fusarium* [10]. Both studies were conducted in northern Nigeria. Therefore, there is paucity of fungal diversity data anchored in molecular techniques from southern Nigeria. Moreover, most of the fungal diversity studies on vended maize, rice, and pupuru relied on data obtained from morphology and/or microscopy alone [2,5,11,12,13,14,15]. Applying phenotyping methods alone for fungal diversity studies have several drawbacks such as lack of precision and species misidentification [16,17]. Irrespective of the available data, there is currently no genera/species-specific based study on the fungal diversity of pupuru anywhere and reliable data on fungal diversity of maize and rice is limited in Nigeria. This creates a major knowledge gap for a fungi surveillance database in the country and, therefore, presents a major hurdle to mitigation efforts.

Under favourable environmental conditions, toxigenic fungi may produce toxic metabolites, such as aflatoxins in maize, rice, and pupuru during pre-harvest phase [18]. Moreover, storing these foods for long periods in non-hermetic devices could further encourage aflatoxin formation [18]. Chronic exposure to aflatoxins results in adverse human health effects such as cancers, stunting in children and death [18,19]. Previously, copious amounts of aflatoxins were quantified in maize from Ondo state, Nigeria [11,20], thus making the state a likely hot-spot for aflatoxin contamination. Yet, there is a paucity of data on aflatoxin contents of vended rice and pupuru from the state. Therefore, this study was carried out to determine the fungal diversity of maize, rice, and pupuru using molecular markers able to identify fungi at a species level and quantify the aflatoxin levels by ELISA.

## 2. Materials and Methods

### 2.1. Study Area

Ondo state is a south-western Nigerian state situated in the Derived savannah agroecological zone. The rainfall pattern is a bimodal distribution averaging between 1000 and 1300 mm per year and the temperature varies from 26 to 38 °C [11,21]. Food crops, such as maize and cassava, are grown mostly by subsistent farmers in the state [22], whereas rice is mostly imported from other states.

### 2.2. Sampling of Foods

A total of 106 food samples consisting of maize (*n* = 46), pupuru (*n* = 20) and rice (*n* = 40) were randomly purchased from major markets (Akure, Ondo, Ore, and Owo) in Ondo state, Nigeria. Samples were purchased between December 2019 and January 2020. In each market, 100 g per sample of a food type was randomly collected from three parts of the vending vessel (basin or bowl) into clean polyethylene bags. The maize and rice samples were ground into fine powder using an electric blender (MX-AC400, Panasonic, Haryana, India), whereas pupuru did not require grinding since it is vended as flour. All ground grains and pupuru were batched in two: batch A for moisture content and mycological analysis and batch B for aflatoxin analysis. Batch A samples were stored at 4 °C and analyzed within 48 h. For batch B, samples of the same food type collected from the same vendor were mixed and stored at −20 °C prior to ELISA analysis. The total number of composite samples from the batch B samples was 42 (maize = 12; pupuru = 10; rice = 20).

### 2.3. Moisture Content Analysis of Ground Grains and Pupuru

The food samples were subjected to moisture analysis by the oven-drying to constant weight method [23]. Five grams of each sample were weighed and dried in a hot air oven at 105 °C. The weight of the samples was measured every hour until constant weight was achieved. Weight measurements per sample were taken in triplicate.

### 2.4. Isolation of Fungi from Ground Grains and Pupuru

Moulds in the ground grains and pupuru samples were isolated by the dilution plating technique [24]. Ten grams of a food sample was diluted in 90 mL of sterile distilled water. The mixture was then homogenized for 2 min on a vortex mixer prior to spread-plating (100 μL) on malt extract agar (MEA; Oxoid, Hampshire, UK). All inoculated plates were incubated at 25 °C for 3 days. In order to enumerate fungi in the food samples, fungal colonies on the plates were counted and reported as colony forming units per gram (CFU/g) of the food sample. Furthermore, distinct colonies on the isolation plates were transferred to freshly prepared MEA plates and incubated at 25 °C for 7 days. Thereafter, pure cultures were prepared on MEA slants in 4 mL vials, overlayed with sterile distilled water, and stored at 25 °C.

### 2.5. Characterization of Fungal Isolates

The isolated moulds were characterized based on morphological characteristics and DNA sequencing. All the strains were cultivated on MEA at 25 °C for 7 days and then assessed for macro- and microscopic character according to the descriptions in appropriate keys [10,25,26,27,28]. Isolates with similar phenotypic characters were grouped and representative isolates from each group were subjected to sequence-based identification.

Molecular analysis was conducted by extracting DNA from the representative isolates grown on MEA at 25 °C for 3–5 days. Parts of the β-tubulin (*BenA*) and/or calmodulin (*CaM*) genes were amplified and subsequently sequenced for the *Aspergillus, Penicillium,* and *Talaromyces* isolates and an ITS barcode sequence was generated for the other fungal isolates. Procedures were as previously described by Houbraken et al. [16,29] and Samson et al. [10]. The generated sequences were compared with sequences present in the National Center for Biotechnology Information (NCBI) database and the curated database of Food and Indoor Mycology department (DTO) housed at the Westerdijk Fungal Biodiversity Institute (WI). All molecularly identified isolates are maintained in the DTO working culture housed at the WI. The newly generated sequences were deposited in GenBank under accession number MZ014549–MZ028006. The potential of the isolates belonging to *Aspergillus* section *Flavi* to biosynthesize aflatoxins was tested in vitro on neutral red desiccated coconut agar (NRDCA) as described by Ezekiel et al. [30].

### 2.6. Aflatoxin Analysis of Ground Grains and Pupuru by Enzyme-Linked Immunosorbent Assay

The concentration of aflatoxins (sum of aflatoxins B and G) present in the ground grains and pupuru samples was determined using a quantitative ELISA kit assay (R4701; RIDASCREEN, Inc., Darmstadt, Germany) following the manufacturer’s instructions. Briefly, all reagents and food samples were allowed to reach ambient temperature. Of each sample, 20 g was mixed with 100 mL 70% methanol extraction solvent. The mixture was homogenized in a shaker (UNISCOPE SM101, Surgifriend, England) for 10 min. The supernatant was carefully decanted and filtered through Whatman No. 1 filter paper. The obtained filtrate (100 μL) was subsequently diluted with distilled water (600 μL).

Exactly 50 μL of aflatoxin standards (0, 0.05, 0.15, 0.45, 1.35, and 4.05 μg/kg) and diluted filtrate was dispensed in duplicates and 50 μL of conjugate was added into each well. Antibody (50 μL) was added to each well, mixed gently by shaking the plate manually prior to incubation for 30 min at ambient temperature. Following incubation, the content of each well was disposed, and the wells washed thrice with wash buffer (250 μL) before drying on an absorbent paper. Aliquots (100 μL) of the substrate/chromogen was added to each well prior to mixing by gently shaking the plates manually and incubated for 15 min at ambient temperature. Thereafter, 100 μL of stop solution was added to each well and the optical densities of the solution in the microtiter plates was read at 450 nm within 30 min using the Microplate reader (LABTRON LMPR-A30, Surrey, UK) [20]. Aflatoxin concentration in each well was calculated from a standard curve plotted using the percentage binding against the total aflatoxin standards. The recovery and limit of detection (LOD) of the ELISA method were 85% and 1.75 μg/kg, respectively.

### 2.7. Data Analysis

All data from this study were analyzed by descriptive statistics using the SPSS Statistics package version 21.0 (SPSS Inc. Chicago, IL, USA). Means for the data on moisture content as well as for the total aflatoxin concentrations in the food types were calculated and tested for significance using the one-way ANOVA (*α* = 0.05). Duncan’s multiple range test was used to separate means. Additionally, Pearson’s correlation coefficient was determined for the relationship between fungal load and moisture content in the ground grains and pupuru.

## 3. Results

### 3.1. Moisture Contents of Ground Grains and Pupuru

The moisture content of the ground grains and pupuru samples ranged between 3.51% and 16.1%. Pupuru has the highest mean moisture level (mean: 11.28 ± 0.35%; range: 9.09–14.6%) followed by rice (mean: 9.44 ± 0.46%; range: 5.08–16.07%) and then maize (mean: 7.72 ± 0.30%; range: 3.51–12.28%) (Table 1). The mean moisture level of pupuru (11.28 ± 0.35%) was significantly (*p* < 0.05) higher than the mean levels recorded for the ground grains.

### 3.2. Incidence of Fungi in Ground Grains and Pupuru

#### 3.2.1. Distribution of Fungi

**Figure 1:**
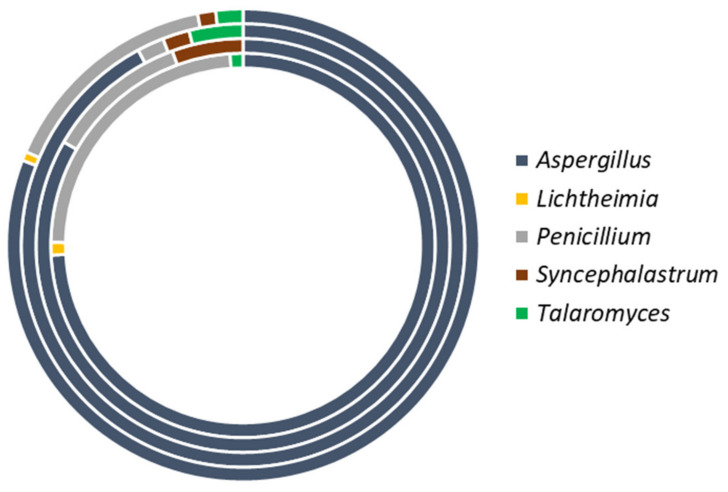
Incidence of fungal genera in ground grains and pupuru vended in markets in Ondo state, Nigeria. Inner ring to outer ring represent maize, pupuru, rice and all foods, respectively.

The fungal load, expressed as Log_10_ colony forming units (CFU)/g, in the foods ranged 2.0–4.8 log_10_ CFU/g, with specific ranges and mean values for each food type as: maize (2.0–4.8 log_10_ CFU/g; 3.90 ± 0.11 log_10_ CFU/g), pupuru (2.0–4.5 log_10_ CFU/g; 2.68 ± 0.20 CFU/g) and rice (2.0–4.6 log_10_ CFU/g; 2.64% ± 0.10 CFU/g). In total, 162 fungal isolates were recovered from 93 (87.7%) of the 106 food samples. The foods were predominated by members of the phylum *Ascomycota* to which *Aspergillus* (80.9%), *Penicillium* (15.4%), and *Talaromyces* (1.9%) belong. The other isolates belong to the *Mucoromycota* (*Syncephalastrum* 1.2%, *Lichtheimia* 0.6%). The occurrence frequency of the isolated fungi is shown in Figure 1.

#### 3.2.2. Species Diversity

**Figure 2:**
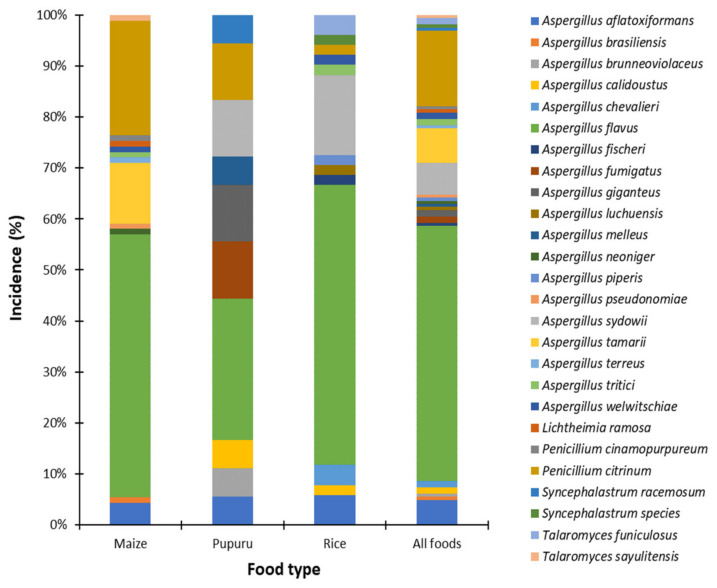
Species diversity of filamentous fungi in ground grains and pupuru vended in markets in Ondo state, Nigeria.

Overall, 26 species were recovered from the ground grains and pupuru samples. Maize and rice both contained 13 different fungal species and 10 different fungal species were recovered from pupuru. *Aspergillus* was the predominant genus and the following species were detected (listed per section, according their prevalence): section *Flavi* (*n* = 101; *A*. *aflatoxiformans*, *A*. *flavus*, *A. tamarii* and *A*. *pseudonomiae*), *Nigri* (*n* = 7; *A*. *brasiliensis*, *A*. *brunneoviolaceus*, *A*. *luchuensis*, *A*. *neoniger*, *A*. *piperis* and *A. welwitschiae*), *Fumigati* (*n* = 3; *A*. *fischeri* and *A*. *fumigatus*), *Clavati* (*n* = 2; *A*. *giganteus*), *Circumdati* (*n* = 1; *A*. *melleus*), *Nidulantes* (*n* = 10; *A. sydowii*), *Terrei* (*n* = 1; *A*. *terreus*), *Candidi* (*n* = 2; *A*. *tritici*), *Usti* (*n* = 2; *A*. *calidoustus*) and *Aspergillus* (*n* = 2; *A*. *chevalieri*). Other species included *Penicillium citrinum* (*n =* 24), *P. cinnamopurpureum* (*n* = 1), *Talaromyces funiculosus* (*n* = 2), *Talaromyces sayulitensis* (*n* = 1)*, Lichtheimia ramosa* (*n* = 1), and *Syncephalastrum racemosum* (*n* = 1). *Aspergillus flavus* was the predominant species occurring in 50% of the food samples. The species diversity of filamentous fungi in the foods is shown in Figure 2 and Supplementary Table S1.

#### 3.2.3. Incidence of Aflatoxigenic and Non-Aflatoxigenic Species of *Aspergillus* Section Flavi

**Figure 3:**
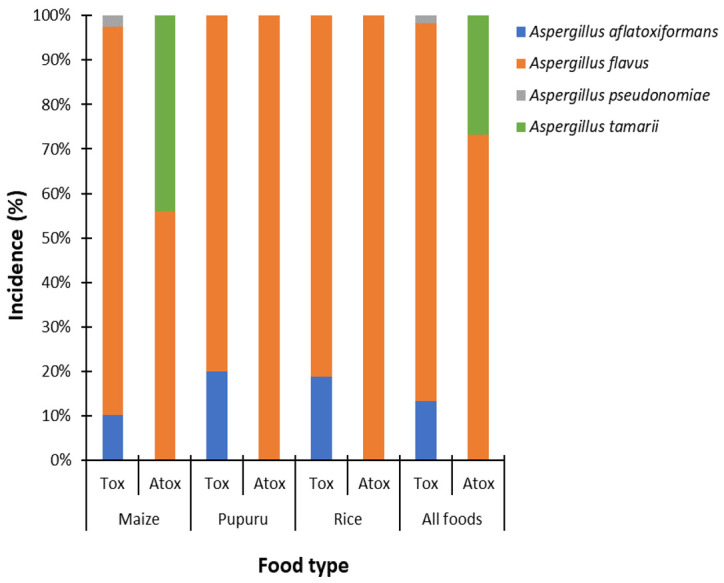
Incidence of aflatoxigenic and non-aflatoxigenic *Aspergillus* section *Flavi* members in foods vended in markets in Ondo state, Nigeria. Tox: aflatoxigenic *Aspergillus* section *Flavi*; Atox: non-aflatoxigenic *Aspergillus* section *Flavi*.

Of the 101 isolates belonging to *Aspergillus* section *Flavi*, 80.2%, 10.9%, 7.9%, and 1.0% were *A. flavus*, *A. tamarii*, *A. aflatoxiformans,* and *A. pseudonomiae*, respectively. About 59% of the 101 isolates were toxigenic on NRDCA, whereas 41% were non-toxigenic. The incidences of aflatoxigenic and non-aflatoxigenic species in the foods are shown in Figure 3 and Supplementary Table S2.

### 3.3. Aflatoxin Levels in Ground Grains and Pupuru Samples

ELISA analysis revealed that 31 (73.8%) of the 42 composite food samples were contaminated with aflatoxins (Table 2). Overall, the total aflatoxin levels in the food samples were in the range of 1.75 to 173.3 μg/kg (mean: 43.1 μg/kg). The intra-assay and inter-assay coefficients of variations were 2.67% and 6.77%. The incidence and levels in each food type were: maize (incidence: 100%; range: 3.50–173.3μg/kg; mean: 100.6 ± 19.3 μg/kg), pupuru (incidence: 40%; range: 1.75–21 μg/kg; mean: 7.9 ± 4.4 μg/kg), and rice (incidence: 75%; range: 1.75–22.8 μg/kg; mean: 6.5 ± 1.6 μg/kg). Only 29.0% of the food samples contained aflatoxins below the EU threshold of 4 μg/kg for total aflatoxins, while 41.9% of the samples exceeded the 10 μg/kg threshold adopted in Nigeria for total aflatoxins.

## 4. Discussion

Post-harvest fungal growth in food is largely influenced by the moisture levels in the foods [9,31]. Generally, the mean moisture contents of the food samples were low (≤11), suggesting they were stored under safe conditions [31,32]. Significantly (*p* < 0.05) higher mean moisture levels were reported in the cassava-based flour (pupuru) compared to the grains (maize and rice). This observation could be attributed to the high-water content in cassava (the raw ingredient of pupuru) when compared to grains as well as the pupuru production process, which involves addition of water. The mean moisture levels recorded for maize and rice (10.4% and 12.5%, respectively) were lower than the values previously reported for maize and rice in Nigeria [2,33] and maize from Ethiopia [34]. Conversely, the mean moisture level in pupuru was similar to the value (11.8%) previously reported in pupuru in Nigeria [7]. There was a weak negative correlation (r = −0.245, *p* < 0.05) between the moisture content in the ground grains and pupuru samples and the fungal load therein. Thus, the low moisture levels in the three sample types examined in the present study suggest that the recovery of fungal propagules from the sample types is rather influenced by contamination along the production chain and during storage of the foods as well as by open vending of unpackaged grains, and not primarily by moisture level [9,35]. To put this into proper context, the food samples were collected from major markets, which are characterized by increased human activities and vehicular movements, such that diverse fungal spores become disseminated and are carried over into the foods.

There was a marked difference in the diversity of species in the three sample types, with higher species diversity (13 species) occurring in maize and rice compared to 10 species found in pupuru. The obvious reason for the lower species diversity in the processed pupuru compared to the grains (maize and rice) is the application of heat during pupuru processing, which has the capacity to eliminate fungal propagules in foods. *Aspergillus* was the predominant genus in the grains and cassava-based flour, occurring in more than three quarter of all the food samples examined. This is consistent with previous reports on the *Aspergillus* dominance in Nigerian foods, such as maize [21,36], rice [4], and garri, a cassava-based product from Nigeria [35]. These data are in contrast to reports on fungal profiles from maize in South Africa [37] and China [38], where *Fusarium* was the predominant genus. In this study, *Fusarium* was not recovered from any of the food samples, which agrees with findings from two previous studies conducted in Nigeria on rice [2] and pupuru [12]. Conversely, this finding from the present study contradicts other available reports [3,34,39,40] indicating several *Fusarium* species in grains, including rice and maize. *Penicillium* was the second most predominant genus in the food samples, occurring in all the sample types and mostly in maize and pupuru. High incidence of *Penicillium* was previously reported in maize from Ethiopia [34] and pupuru from Nigeria [12]. Other fungal genera recovered from the sample types include *Talaromyces* (only recovered from the grains) as well as *Syncephalastrum* (from pupuru and rice) and *Lichtheimia* (found only in maize). The latter genus was previously known as *Absidia* and has been reported in Chinese and Brazilian maize [41] but is, to the best of our knowledge, reported for the first time in Nigerian maize.

In this study, *A. flavus* was the predominant species in the grains and pupuru samples, occurring in 50% of all the samples. The predominance of *A. flavus* in the foods is supported by previous reports from Nigeria [4,42,43] and elsewhere [44,45]. *Aspergillus aflatoxiformans* was recovered from all the sample types, but mostly in the grains. This agrees with a previous report from Nigeria, wherein *A. aflatoxiformans* predominated in grains including maize and rice [3]. *Aspergillus aflatoxiformans* (previously, wrongly classified as *A. parvisclerotigenus*) has been reported in cassava from neighbouring Benin Republic [46], as well as dried mushroom and peanuts from Nigeria [47,48]. However, the occurrence of *A. aflatoxiformans* from any Nigerian cassava-based food has not been documented until now. Two other members of *Aspergillus* section *Flavi* (*A. tamarii* and *A. pseudonomiae*) were only found in maize, which is consistent with previous reports [3,45,49]. Of the *Aspergillus* section *Flavi* isolates tested for toxigenicity in vitro on NRCDA, more than one half of the *A. flavus* strains and all of the *A. aflatoxiformans* and *A. pseudonomiae* exhibited toxigenic potential on NRDCA. Aflatoxin production is a chemotaxonomic signature in the *Aspergillus* section *Flavi* group, especially among strains of *A. flavus*, *A. aflatoxiformans,* and *A. pseudonomiae* [50]. Consequently, the recovery of toxigenic strains in the present study may imply that frequent ingestion of these foods is a contributory factor to the high aflatoxin exposure recorded in Nigeria [51,52,53,54,55].

The fungal species belonging to *Aspergillus* section *Nigri* recovered from the food samples include *A. neoniger*, *A. brasiliensis*, *A. brunneoviolaceus*, *A. luchuensis*, *A. piperis*, and *A. welwitschiae* [56]. *Aspergillus brunneoviolaceus* was only recovered from pupuru. This observation agrees with the report on *A. brunneoviolaceus* in garri, another cassava-based product, in Nigeria [35] and suggests an association of this species with cassava (products). Other members of the section *Nigri* were recovered only from the grains. *Aspergillus welwitschiae* was only recovered from maize, which is in agreement with a previous report from USA and Italy [57]. Similarly, *A. brasiliensis* and *A. neoniger* were recovered from maize, whereas *A. luchuensis* and *A. piperis* were found in rice. *Aspergillus fumigatus* (section *Fumigati*), a notorious opportunistic pathogen [58], was only recovered from pupuru. The other member of the *Aspergillus* section *Fumigati*, *A. fischeri*, was found in rice. Other members of the *Aspergillus* genera found only in pupuru include *A. calidoustus* in *Aspergillus* section *Usti* [59], A. giganteus in *Aspergillus* section *Clavati* [60], and *A. melleus* in *Aspergillus* section *Circumdati* [61], which are reported for the first time in Nigerian dried cassava-based food.

*Penicillium citrinum*, a known citrinin producer [62], was recovered from all the sample types. Recent reports found this fungus in cocoa and dried ready-to-eat foods from Nigeria [35,63], with the strains producing copious amounts (up to 372 mg/kg) of citrinin [63]. Furthermore, high levels (16,800 µg/kg and 51,195 µg/kg) of citrinin were previously quantified in Nigerian maize [64,65]. Citrinin is a nephrotoxic mycotoxin [66]. Putting findings from the aforementioned reports together with the high incidence of *P. citrinum* in this study, there is an urgent need for mitigation efforts targeted at reducing *P. citrinum*, which indirectly reduces citrinin levels, in Nigerian foods. Other notable fungal species include *P. cinamopurpureum, L. ramosa*, *T. funiculosus,* and *T. sayulitensis* recovered only from the grains and *Syncephalastrum racemosum* found only in pupuru. *Talaromyces sayulitensis* is mostly associated with maize [67], which agrees with findings of the present study. *Lichtheimia ramosa* is a soil fungus that causes mucormycosis especially in immunocompromised individuals [68]. *Syncephalastrum racemosum* can cause onychomycosis [69] and this fungus has been previously reported in cocoa seeds in Nigeria [70], but its occurrence in pupuru was not yet been documented to date. Major limitations of this study are the use of MEA for fungal isolation and the incubation of fungal culture plates for three days, thus precluding the recovery of slow growing fungi during isolation. However, the main focus was to enumerate mostly toxigenic fungi (*Aspergillus* and *Penicillium*) from the food samples as such data from the study is relevant.

In order to ascertain the aflatoxicological safety of the grains and cassava-based flour, the levels of aflatoxin in the foods were determined. The focus on aflatoxin determination amidst many other mycotoxins emanated from its status as the most toxicologically important mycotoxin due to its categorization as Class 1 carcinogen [71]. In the present study, 73.8% of the examined composite food samples contained aflatoxins. This is consistent with previous reports from Nigeria [20,72] and Kenya [73] that applied ELISA protocols in food analysis and reported high aflatoxin incidence > 70%. The detection of aflatoxin in the pupuru samples agrees with a previous report from a market in a town close to the study locations, where aflatoxins were detected in 70% of pupuru samples albeit at very low concentrations [74]. About 71% of the food samples exceeded the 4 μg/kg threshold set by the European Union (EU) for total aflatoxins [75]. The proportion of samples exceeding the EU limit is high and calls for more caution and the need to set in place critical intervention measures to limit aflatoxin levels in the foods. This is crucial for cereals such as maize and rice, which have the potential to be aggregated at the local market and exported to the EU. Overall, the high levels of aflatoxins quantified in the food samples agree with the data obtained for the high incidence of toxigenic *Aspergillus* species recovered from the food samples in the present study. It is likely that poor storage practices, which are very common in Nigeria [9], contributed to the high aflatoxin levels in the food samples. Nevertheless, a more robust mycotoxin surveillance study anchored in aa liquid chromatography-based method is required to understand the actual extent of aflatoxin contamination in the foods vended in Nigeria.

## 5. Conclusions

This study provides snapshot data on the fungal diversity and aflatoxin contents of grains and pupuru vended in open markets in Ondo state, Nigeria. Diverse fungal species and high aflatoxin levels were found in the examined foods, suggesting the possible influence of poor handling, processing, and storage on the contamination of the foods. Urgent mitigation efforts are required to limit toxigenic fungal and aflatoxin contamination of these foods in the country. We therefore recommend proper storage of foods in hermetic devices, such as Purdue Improved Crop Storage (PICS) bags and metal silos, as detailed by Ayeni et al. [9]. In addition, households are advised to properly sort and filter bad grains before preparing foods.

## Figures and Tables

**Table 1:** Moisture contents of maize, pupuru and rice vended in Ondo state, Nigeria.

Food Type	N	Mean (%) ± SE	Range (%)	
			Min	Max
Maize	46	7.72 ± 0.30 ^c^	3.51	12.3
Pupuru	20	11.3 ± 0.35 ^a^	9.10	14.6
Rice	40	9.44 ± 0.46 ^b^	5.08	16.1
Total	106	9.04 ± 0.26	3.51	16.1

N = number of samples. Means with same superscript alphabet (a–c) in a column do not differ significantly (α = 0.05).

**Table 2:** Incidence of aflatoxins in 42 ground grains and pupuru from Ondo state, Nigeria.

Food Type	N	%	Incidence (%) of Contaminated Food Samples					Mean (μg/kg) ± SE	Range (μg/kg)	
			≤4 μg/kg *	≤ 10 μg/kg	≤20 μg/kg **	>20–<100 μg/kg	≥100 μg/kg		Min	Max
Maize	12	100	8.3	16.7	25.0	16.7	58.3	101 ± 19.3	3.5	173.3
Pupuru	10	40.0	50.0	75.0	75.0	25.0	0.00	7.9 ± 4.4	1.75	21
Rice	20	75.0	40.0	86.7	93.3	6.7	0.00	6.5 ± 1.6	1.75	22.8
Total	42	73.8	29.0	58.1	64.5	12.9	22.6	43.1 ± 11.1	1.75	173.3

N = number of samples; % = percentage contaminated samples; * European Union regulatory limit for total aflatoxins in foods; ** United States Food and Drug Administration regulatory limit for total aflatoxins in foods.

## Data Availability

Not applicable.

## Supplementary Materials

The following are available online at https://www.mdpi.com/article/10.3390/jof7080635/s1, Table S1: Numerical values for fungal species diversity in the ground grains and pupuru samples, and Table S2: Numerical values for toxigenicity of members of *Aspergillus* section *Flavi* in the foods.
